# Canine peripheral non-conventional TCRαβ^+^ CD4^-^CD8α^-^ double-negative T cells show T helper 2-like and regulatory properties

**DOI:** 10.3389/fimmu.2024.1400550

**Published:** 2024-05-21

**Authors:** Martina Protschka, Daniela Di Placido, Peter F. Moore, Mathias Büttner, Gottfried Alber, Maria Eschke

**Affiliations:** ^1^ Institute of Immunology, Center for Biotechnology and Biomedicine, Faculty of Veterinary Medicine, Leipzig University, Leipzig, Germany; ^2^ Department of Pathology, Microbiology and Immunology, School of Veterinary Medicine, University of California, Davis, Davis, CA, United States

**Keywords:** dog, canine TCRαβ^+^ T cells, CD4^-^CD8α^-^ double-negative, CD4^+^CD8α^+^ double-positive, non-conventional T cells, Th2 Cells, Treg cells, peripheral blood

## Abstract

The dog is an important companion animal and also serves as model species for human diseases. Given the central role of T cells in immune responses, a basic understanding of canine conventional T cell receptor (TCR)αβ^+^ T cells, comprising CD4^+^ single-positive (sp) T helper (Th) and CD8α^+^ sp cytotoxic T cell subsets, is available. However, characterization of canine non-conventional TCRαβ^+^ CD4^+^CD8α^+^ double-positive (dp) and TCRαβ^+^ CD4^−^CD8α^−^ double-negative (dn) T cells is limited. In this study, we performed a comprehensive analysis of canine dp and dn T cells in comparison with their conventional counterparts. TCRαβ^+^ T cells from peripheral blood of healthy dogs were sorted according to their CD4/CD8α phenotype into four populations (i.e. CD4^+^ sp, CD8α^+^ sp, dp, and dn) and selected surface markers, transcription factors and effector molecules were analyzed *ex vivo* and after *in vitro* stimulation by RT-qPCR. Novel characteristics of canine dp T cells were identified, expanding the previously characterized Th1-like phenotype to Th17-like and Th2-like properties. Overall, mRNA expression of various Th cell-associated cytokines (i.e. *IFNG, IL17A, IL4, IL13*) in dp T cells upon stimulation highlights their versatile immunological potential. Furthermore, we demonstrated that the CD4^-^CD8α^-^ dn phenotype is stable during *in vitro* stimulation. Strikingly, dn T cells were found to express highest mRNA levels of type 2 effector cytokines (*IL4, IL5, and IL13*) upon stimulation. Their strong ability to produce IL-4 was confirmed at the protein level. Upon stimulation, the percentage of IL-4-producing cells was even higher in the non-conventional dn than in the conventional CD4^+^ sp population. Constitutive transcription of *IL1RL1* (encoding IL-33Rα) further supports Th2-like properties within the dn T cell population. These data point to a role of dn T cells in type 2 immunity. In addition, the high potential of dn T cells to transcribe the gene encoding the co-inhibitory receptor CTLA-4 and to produce the inhibitory cytokine IL-10 indicates putative immunosuppressive capacity of this population. In summary, this study reveals important novel aspects of canine non-conventional T cells providing the basis for further studies on their effector and/or regulatory functions to elucidate their role in health and disease.

## 1 Introduction

Dogs develop a number of spontaneous immune-mediated disorders, such as autoimmune diseases (1) and allergies (2), as well as cancers (3). Similarities to these complex diseases in humans make the dog particularly interesting as an animal model. Given the importance of T cells in health and disease, comprehensive knowledge of canine T cells is essential to elucidate pathogenesis mechanisms, to develop new treatment strategies, and to appropriately use dogs as a model for these strategies.

Besides the conventional TCRαβ^+^ T cells expressing either CD4 or CD8α as co-receptor (i.e. CD4^+^ single-positive (sp) and CD8α^+^ sp T cells), dogs harbor non-conventional TCRαβ^+^ CD4^+^CD8α^+^ double-positive (dp) (4) and TCRαβ^+^ CD4^-^CD8α^-^ double-negative (dn) T cells (5). Whereas conventional cytotoxic CD8 T cells mainly express the CD8αβ heterodimer, canine non-conventional dp T cells predominantly express the CD8αα homodimer (6, 7). Due to their special co-receptor equipment, dp T cells possess unique immunological potential. In humans, dp T cells have been shown to be functionally relevant in cancer, infections and autoimmune diseases (8, 9), while in mice, they have been found to accumulate at the site of injection following subcutaneous vaccination (10). In dogs, increased frequencies of CD4^+^CD8α^+^ dp T cells were reported after infection with *Ehrlichia chaffeensis* (11) and in the context of canine leishmaniasis (12). As shown by our group, mature extrathymic TCRαβ^+^ dp T cells comprise about 2% of circulating T cells in healthy dogs and are characterized by an activated phenotype indicated by expression of CD25 and MHC-II (6, 13). Furthermore, consistent with their effector memory phenotype, these cells have a strong IFN-γ production capability (6, 7).

Whereas dn T cells account for only 1–3% of all T cells in the peripheral blood of mice and humans (14, 15), in dogs, approximately 15% of all circulating TCRαβ^+^ T cells are double-negative (5). This substantial population of non-conventional T cells is characterized by a remarkably high expression of CD25. CD25 is either co-expressed with FoxP3 (reminiscent of a regulatory phenotype), or without FoxP3 (reminiscent of an effector phenotype). In addition, subsets expressing IFN-γ, GATA-3, or IL-17A suggest properties of conventional type 1 T helper (Th1), Th2, and Th17 cells, respectively (5).

The recently reported increase of dn T cells after allergen desensitization, as well as the greatly increased frequency of dp T cells in dogs with adverse food reactions (16) indicates that canine non-conventional T cells can be involved in immune responses *in vivo*. However, knowledge about these cells is still limited.

To gain deeper insight into their phenotype(s) and functional potential, we performed a comprehensive *ex vivo* and *in vitro* analysis of canine peripheral blood dp and dn T cells in comparison with their conventional CD4^+^ sp and CD8α^+^ sp counterparts.

While dp T cells have versatile immunological potential, our results point to a stable dn phenotype and highlight a remarkable Th2-like potential of dn T cells. Furthermore, their high ability to express inhibitory molecules indicates putative immunosuppressive capacity of canine dn T cells.

## 2 Materials and methods

### 2.1 Dogs, blood samples

Peripheral blood was taken by venipuncture of the *vena cephalica antebrachii* into heparinized collection tubes (BD Vacutainer^®^, 10 ml, Li-Heparin 17 IU/ml Becton Dickinson, Heidelberg, Germany) from healthy experimental Beagle dogs (7 female, 4 male, age: 4-10 years) of the Faculty of Veterinary Medicine (Leipzig University, Leipzig, Germany). The number of dogs used for individual analyses is indicated in the figure legends. All dogs received routine vaccinations against canine distemper, rabies, canine infectious hepatitis, parvovirus infection, parainfluenza, and leptospirosis. The study was authorized by the Saxony State Office (*Landesdirektion Sachsen*) in Leipzig, Germany (approval numbers: DD24.1-5131/444/30; DD24.1-5131/468/16).

### 2.2 Isolation of canine peripheral blood mononuclear cells

PBMC were isolated by density gradient centrifugation. Briefly, blood was diluted with phosphate buffered saline (PBS) at a ratio of 1:1, layered above Biocoll Separating Solution (density 1077 g/l, Biochrom AG, Berlin, Germafny) and centrifuged at 500g for 30 min at room temperature (minimal acceleration and braking). PBMC at the interphase were harvested into PBS and centrifuged at 500g for 10 min at RT. After another washing step with PBS, remaining erythrocytes were lysed by incubation in 150 mM NH_4_Cl, 8 mM KHCO_3_, 2 mM EDTA (pH 7.0) for 5 min at RT. The reaction was stopped by addition of PBS containing 3% fetal bovine serum (FBS, Thermo Fisher Scientific, Carlsbad, USA; and PAN-Biotech, Aidenbach, Germany). After washing with PBS, PBMC were counted in Trypan blue (Sigma-Aldrich, Taufkirchen, Germany) using a hemocytometer (Laboroptik, Lancing, UK).

### 2.3 Fluorescence-activated cell sorting of conventional and non-conventional TCRαβ^+^ T cell subpopulations

**Figure 1:**
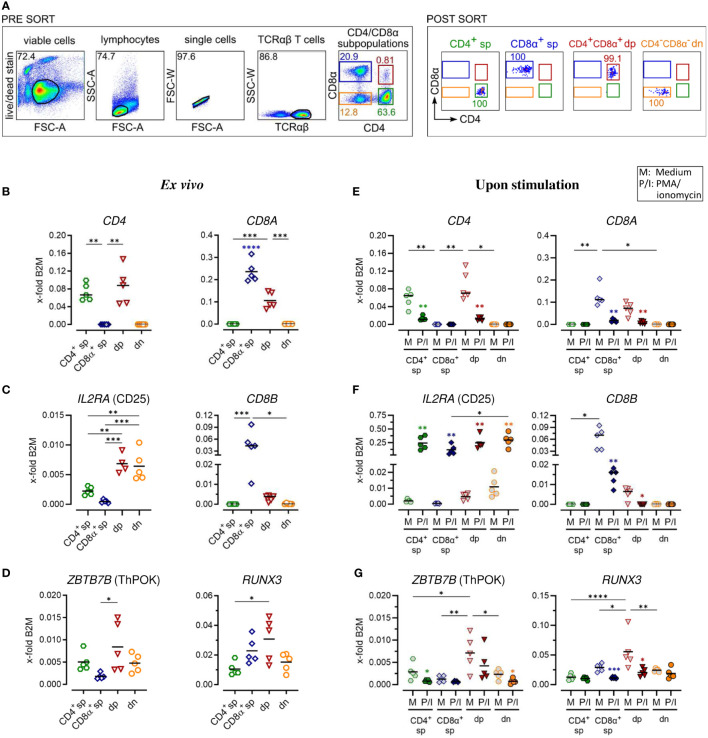
Gene expression analysis of purified canine peripheral conventional and non-conventional TCRαβ^+^ T cell subpopulations reveals stability of the CD4^-^CD8α^-^ double-negative phenotype after PMA/Ionomycin stimulation. **(A)** Strategy for isolation of indicated CD4/CD8α TCRαβ^+^ subpopulations (sp: single-positive, dp: double-positive, dn: double-negative) by fluorescence-activated cell sorting and the purity (percentages) of the sorted populations are shown (representative data of one dog). **(B–G)** These populations were analyzed by RT-qPCR. Expression of indicated genes *ex vivo* **(B–D)** and after 5 h of medium (M) incubation or stimulation with PMA/ionomycin (P/I) **(E–G)** defines the sp/dp/dn phenotype of sorted T cell subpopulations. Note that the expression of ThPOK (which is known to be associated with the gene program of CD4 T helper cells) and Runx3 (which establishes the gene program characteristic of cytotoxic CD8 T cells) is significantly higher in CD4^+^CD8α^+^ dp T cells compared to CD8α^+^ sp and CD4^+^ T cells, respectively. **(D, G)**. Each dot represents one individual dog analyzed separately in independent experiments (n=4 or 5). Depending on normal distribution, data sets are presented with the mean or median (horizontal bars). Statistical differences between subpopulations are marked with lines. Additionally, in **(E–G)**, statistical significance of stimulation-induced effects was calculated for each subpopulation by direct comparison of M versus the P/I equivalent (colored asterisks without lines). (* p < 0.05; ** p < 0.01; *** p < 0.001; **** p < 0.0001). Blue asterisks in **(B)** indicate significantly increased expression of CD8A in the purified CD8A sp population compared to all other populations. B2M, Beta-2-Microglobulin (reference gene for normalization).

All incubation steps performed to stain cells for fluorescence-activated cell sorting were performed for 15-20 min on ice in the dark. First, PBMC were incubated with the fixable viability dye eFluor 780 (Thermo Fisher Scientific, Carlsbad, USA) according to the manufacturer’s protocol to discriminate viable from dead cells. The subsequent staining steps were preceded by washing with PBS, 3% FCS. To block binding of Fc receptors, cells were preincubated for 5 min with a mixture of heat-inactivated normal serum derived from dog and rat (each 15% in PBS) before addition of the anti-canine TCRαβ (clone CA15.8G7) hybridoma supernatant (**Table 1**) and further incubation. In the next incubation step, a PerCP/Cy5.5-conjugated goat-anti-mouse IgG secondary antibody (Biolegend, San Diego, USA) was used for detection of TCRαβ. Finally, PBMC were incubated with FITC-conjugated anti-canine CD4 (clone YKIX302.9) and APC-conjugated CD8α (clone YCATE55.9) antibodies (**Table 1**). Stained cells were applied over a 30 µm filter (Sysmex, Norderstedt, Germany) and immediately sorted using a BD FACSAria™ III Cell Sorter (Becton Dickinson, Heidelberg, Germany). The sorting strategy is shown in **Figure 1A**. After exclusion of dead cells and doublets, TCRαβ^+^ T cells from the lymphocyte gate were selected and CD4^+^ single-positive (sp), CD8α^+^ sp, CD4^bright^CD8α^bright^ double-positive (dp), and CD4^-^CD8α^-^ double-negative (dn) T cell subpopulations were sorted with a purity >99% (Re-analysis using the FlowJo™10 software (Treestar Inc., Ashland, OR, USA).

**Table 1:** Primary antibodies used for fluorescence-activated cell sorting and flow cytometry analysis.

Antigen	Clone	Target species	Isotype	Source	Conjugate
TCRαβ	CA15.8G7	canine	mouse IgG1	Leukocyte Antigen Biology Laboratory, Davis, USA	unconjugated hybridoma supernatant
CD4	YKIX302.9	canine	rat IgG2a	Thermo Fisher Scientific, Carlsbad, USA Bio-Rad, Munich, Germany	APC FITC
CD8α	YCATE55.9	canine	rat IgG1	Thermo Fisher Scientific, Carlsbad, USA Bio-Rad, Munich, Germany	APC PerCP-eFluor 710
CD25	P4A10	canine	mouse IgG1	Thermo Fisher Scientific, Carlsbad, USA	PE
IL-4*	unknown	canine	mouse IgG2a	Canine IL-4 Duoset ELISA detection antibody; R&D Systems, Minneapolis, USA	Biotin
IL-10*	unknown	canine	mouse IgG1	Canine IL-10 Duoset ELISA detection antibody; R&D Systems, Minneapolis, USA	Biotin
IFN-γ	CC302	bovine**	mouse IgG1	Bio-Rad, Munich, Germany	FITC

*antibodies were used in different samples.

**cross-reactive with canine (17).

### 2.4 Stimulation of sorted TCRαβ^+^ T cell subpopulations and whole PBMC

Isolated TCRαβ^+^ T cell subpopulations or whole PBMC were seeded at a density of 5x10^5^ per well in IMDM medium with L-Glutamine and 25 mM HEPES (PAA Laboratories, Cölbe, Germany) supplemented with 10% FBS (Thermo Fisher Scientific, Carlsbad, USA), 100 U/ml penicillin, and 100 μg/ml streptomycin (both purchased from PAA Laboratories) into 96-well round bottom cell culture plates (Techno Plastic Products AG, Trasadingen, Switzerland) and rested over night at 37°C and 5% CO_2_. Then, cells were polyclonally stimulated for 5 hours at 37°C with phorbol myristate acetate and Ionomycin (P/I) (both: 0.22 µg/ml; Sigma-Aldrich, Taufkirchen, Germany). Medium incubation (M) served as negative control. For subsequent intracellular cytokine staining, 5 µg/ml Brefeldin A (BrefA) (Enzo Life Sciences, New York, USA) was added to the culture.

### 2.5 RNA isolation, reverse transcription and real-time PCR analysis

**Figure 2:**
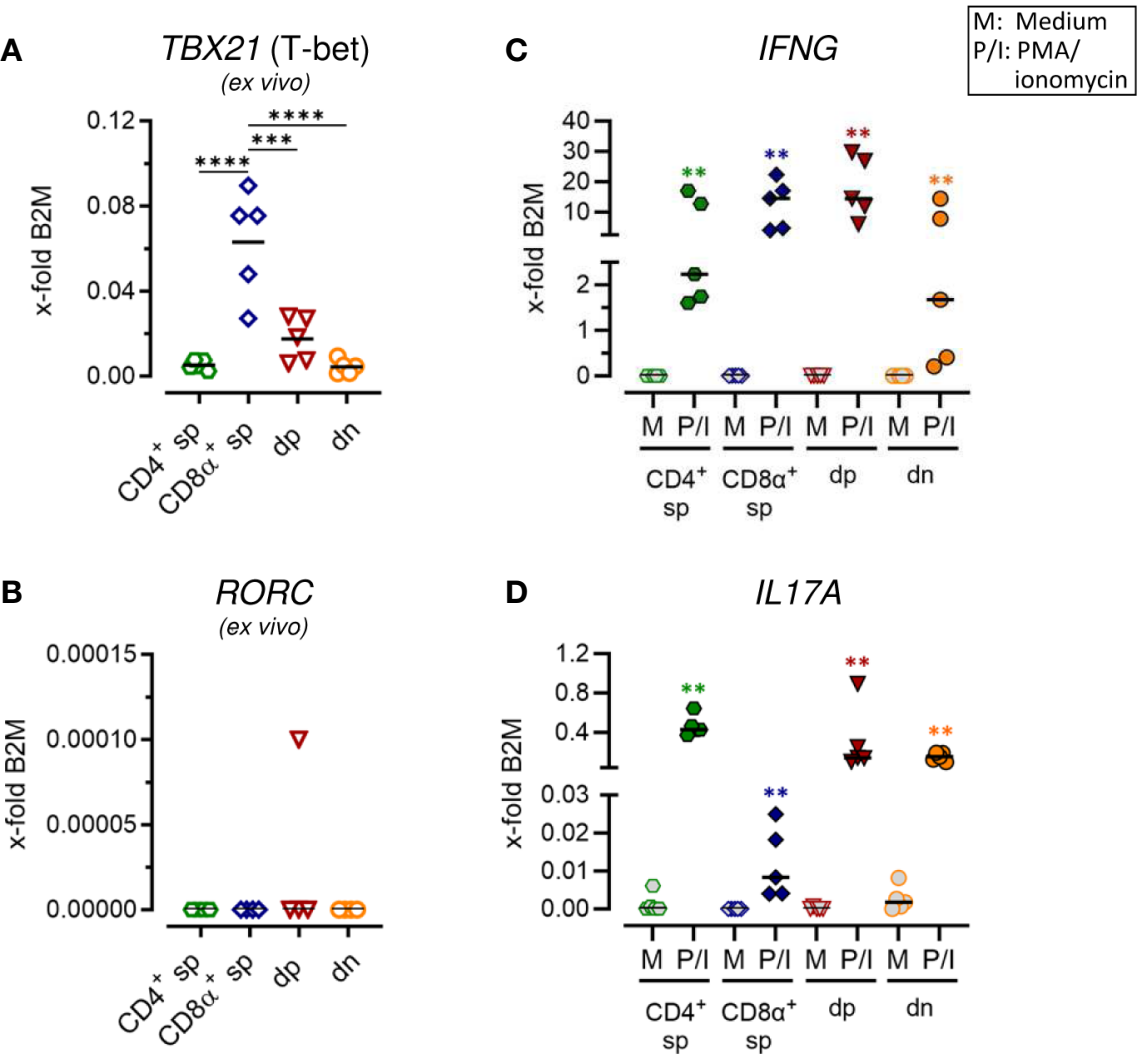
Canine CD4^+^CD8α^+^ double-positive T cells comprise T helper (Th) 1 and Th17 expression properties. **(A–D)** Sorted TCRαβ^+^ T cell subpopulations, as shown in **Figure 1A**, were analyzed for expression of indicated genes *ex vivo* **(A, B)** or after 5 h of medium (M) incubation or stimulation with PMA/ionomycin (P/I) **(C, D)** (sp, single-positive; dp, double-positive; dn, double-negative). Each dot represents one individual dog analyzed separately in independent experiments (n=5). Depending on normal distribution, data sets are presented with the mean or median. Statistical differences between subpopulations are marked with lines. Additionally, in **(C, D)**, statistical significance of stimulation-induced effects was calculated for each subpopulation by direct comparison of M versus the P/I equivalent (colored asterisks without lines). (** p < 0.01; *** p < 0.001; **** p < 0.0001).

**Figure 3:**
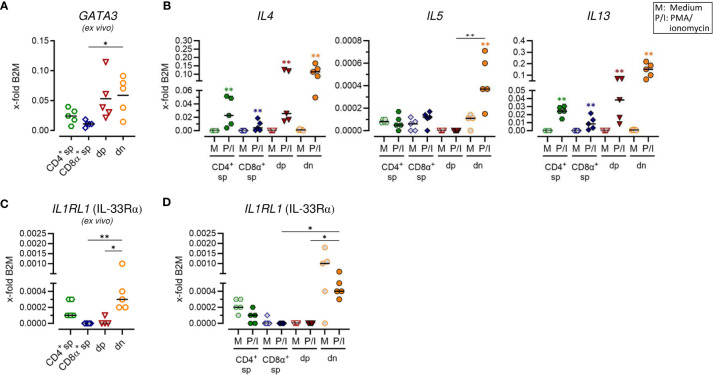
Canine CD4^+^CD8α^+^ double-positive and particularly CD4^-^CD8α^-^ double-negative T cells have a remarkable type 2 potential. **(A–D)** TCRαβ^+^ T cell subpopulations sorted as shown in **Figure 1A** were analyzed for expression of indicated type 2 immune response-related genes *ex vivo* **(A, C)** and/or after 5 h of medium (M) incubation or stimulation with PMA/ionomycin (P/I) **(B, D)** (sp: single-positive, dp: double-positive, dn: double-negative). Each dot represents one individual dog analyzed separately in independent experiments (n=5). Depending on normal distribution, data sets are presented with the mean or median. Statistical differences between subpopulations are marked with lines. Additionally, in **(B, D)**, statistical significance of stimulation-induced effects was calculated for each subpopulation by direct comparison of M versus the P/I equivalent using the Mann-Whitney test (colored asterisks without lines). (* p < 0.05; ** p < 0.01).

**Figure 4:**
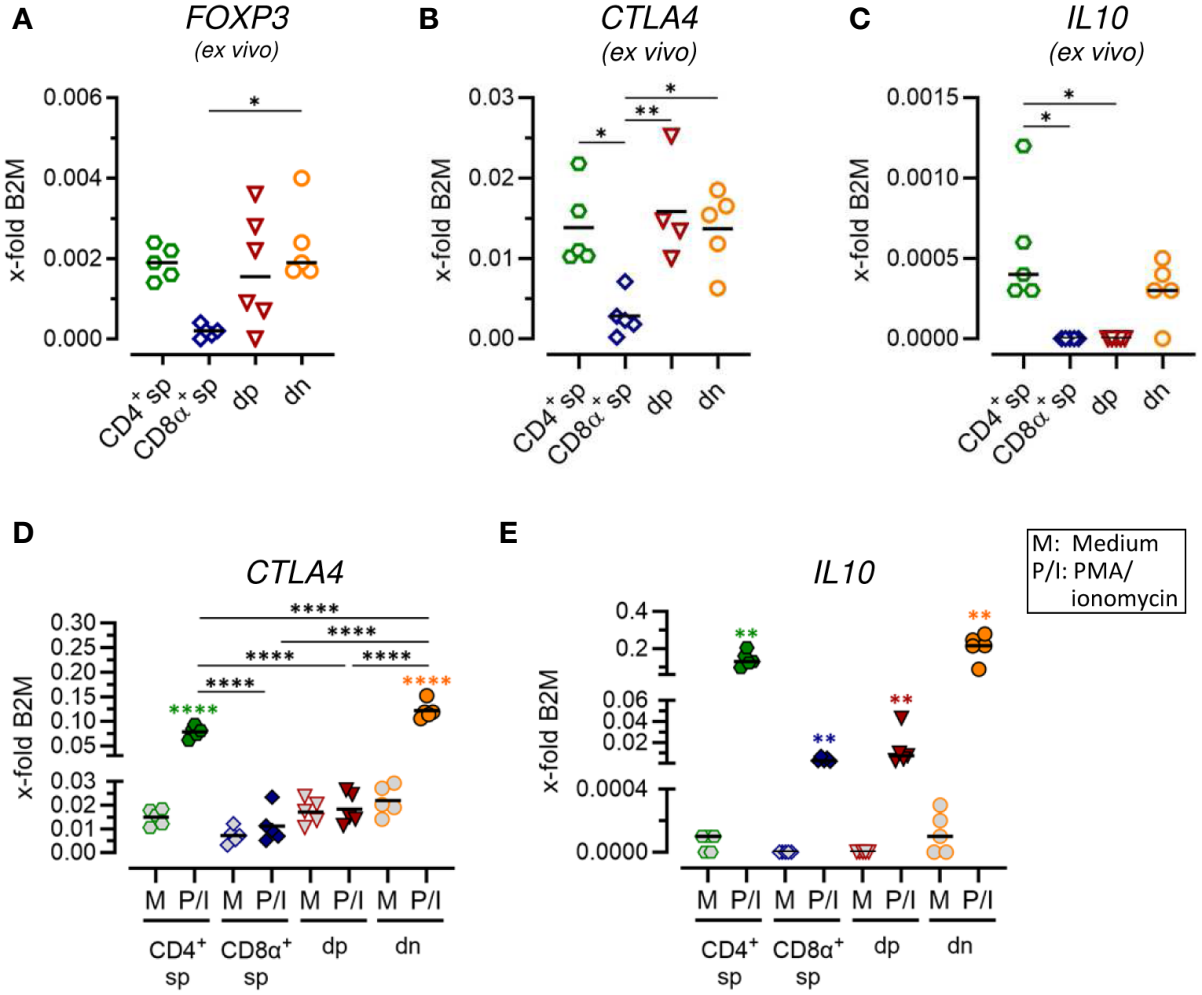
Constitutive expression of key regulatory markers and stimulation-induced upregulation of inhibitory effector molecules indicate immunosuppressive potential of CD4^-^CD8α^-^ double-negative T cells. **(A–E)** TCRαβ^+^ T cell subpopulations sorted as shown in **Figure 1A** were analyzed for expression of indicated Treg-related genes *ex vivo* **(A–C)** or after 5 h of medium (M) incubation or stimulation with PMA/ionomycin (P/I) **(D, E)** (sp, single-positive; dp, double-positive; dn, double-negative). Each dot represents one individual dog analyzed separately in independent experiments (n=5). Depending on normal distribution, data sets are presented with the mean or median. Statistical differences between subpopulations are marked with lines. Additionally, in **(D, E)**, statistical significance of stimulation-induced effects was calculated for each subpopulation by direct comparison of M versus the P/I equivalent (colored asterisks without lines). (* p < 0.05; ** p < 0.01; **** p < 0.0001).

Total RNA from sorted TCRαβ^+^ T cell subpopulations was isolated using 1 ml RNA-Solv^®^ Reagent (Omega Bio-tek, Norcross, USA) following manufacturer instructions. To increase the RNA yield, 2 µl glycogen (Thermo Fisher Scientific, Vilnius, Lithuania) were added per sample. The concentration of the RNA was determined spectrophotometrically (NanoDrop, PEQLAB, Erlangen). DNA was digested with one unit DNase (Thermo Fisher Scientific)/µg RNA for 30 min at 37°C. For isolation of poly(A)+ RNA, an additional ethanol precipitation was conducted with 1/8 volume RNase-free 3 M NaOAc, pH 5.7. Reverse transcription was performed using the ‘High Capacity cDNA Reverse Transcription Kit’ (Applied Biosystems, Foster City, USA), containing RiboLock RNase inhibitor (Thermo Fisher Scientific), and a mixture of oligo (dT)18 (Thermo Fisher Scientific) and random hexamer primers in a PTC-200 (MJ Research, Watertown, USA). Specific primers were designed using the primer-BLAST Software (National Center for Biotechnology Information, Bethesda, USA) and synthesized by Metabion (Planegg, Germany) (**Table 2**). The primers for *IL2RA* (CD25), *IL4*, and *IL5* were commercially available (Bio-Rad, Munich, Germany). Real-time PCR with SYBR^®^ Green detection was performed using the iTaq Universal SYBR^®^ Green Supermix (Bio-Rad, Munich, Germany) and a CFX Connect real time PCR detection system (Bio-Rad). Specificity was tested by melting curve analysis and an agarose gel electrophoresis (LE agarose, Biozym, Hess. Oldendorf; Germany). Only primers resulting in a single peak in melting curve analysis and a single band with the expected fragment size in gel electrophoresis were used. RT-qPCR efficiencies were calculated with the CFX Maestro Software version 2.2 (Bio-Rad) from standard curves generated by serial dilutions of appropriate cDNA samples (2-fold dilutions, 8 point-curve, conducted in duplicates). The efficiency of all reactions ranged between 90 and 110%. Beta-2-microglobulin (*B2M*) and succinate dehydrogenase subunit A (*SDHA*), were used as reference genes of different functional classes with stability verified using the CFX Maestro software based on the GeNorm algorithm (18) (**Supplementary Table 1**). The same amount of cDNA was used for analysis of expression of the respective reference gene and target genes from the same sample. Relative quantification of the transcripts was done by the 2^(-ΔCt)^ method (19, 20). Results obtained with B2M as reference gene are shown in **Figures 1**–**4**, those normalized to SDHA in **Supplementary Figures 1**–**4**.

**Table 2:** Primers used for real-time PCR.

Gene name	Primer sequence	GenBank ACCN*	Position	Exon	Product length
B2M_for	5´-CTCATCCTCCTCGCTGCGT-3´	NM_001284479.1	49-67	1	84 bp
B2M_rev	5´-ATTCTCTGCTGGGTGTCGTGA-3´	NM_001284479.1	132-112	2	
CD4_for	5´-CGGTATGCTGGTTCTGGAAT-3´	NM_001003252.1	1044-1063	6	118 bp
CD4_rev	5´-GCACCTCACAGGTCAGATTG-3´	NM_001003252.1	1161-1142	7	
CD8A_for	5´-CGTCCTTCTCCTGTCACTGG-3´	NM_001002935.2	630-649	4	150 bp
CD8A_rev	5´-GCCACACAGGATCCATCTCC-3´	NM_001002935.2	779-760	6	
CD8B_for	5´-CAAGAAGAGAGTGTGCCGGT-3´	XM_038691486.1	542-561	3	118 bp
CD8B_rev	5´-CACACCCAAGGACACCAGTA-3´	XM_038691486.1	659-640	4	
CTLA4_for	5´-GGATCCTTGCAGCAGTCAGT-3´	NM_001003106.1	553-572	3	140 bp
CTLA4_rev	5´-ACATTCTGGCTCAGTTGGGG-3´	NM_001003106.1	692-673	4	
FOXP3_for	5´-ACAGCACATTCCCAGAGTTCTT-3´	NM_001168461.1	1033-1054	8/9	130 bp
FOXP3_rev	5´-TTGAGTGTCCGCTGCTTCTC-3´	NM_001168461.1	1162-1143	10	
GATA3_for	5´-GCGAACTGTCAAACCACCAC-3´	XM_038660403.1	973-992	5	147 bp
GATA3_rev	5´-TCGGTTTCTGGTCTGGATGC-3´	XM_038660403.1	1119-1100	6	
IFNG_for	5´-GAAATGGAGAGAGGAGAGTGACA-3´	NM_001003174.1	276-298	2/3	239 bp
IFNG_rev	5´-TGAGTTCATTTATCGCCTTGC-3´	NM_001003174.1	514-494	4	
IL10_for	5´-GCAGGTGAAGAGCGCATTTAG-3´	NM_001003077.1	430-450	4	150 bp
IL10_rev	5´-GCCATCCTGGGTGTTTTGTTC-3´	NM_001003077.1	579-559	5	
IL13_for	5´-GAGCTGGTCAACATCACCCA-3´	NM_001003384.1	154-173	1	147 bp
IL13_rev	5´-CCTCTGGGTCCTTTGGATGG-3´	NM_001003384.1	300-281	3	
IL17A_for	5´-AACTCCAGAAGGCCCTCAGAT-3´	NM_001165878.1	185-205	2	107 bp
IL17A_rev	5´-CACTTCGCCTCCCAGATCAC-3´	NM_001165878.1	291-272	3	
IL1RL1_for	5´-AATTACAGCGTGACAGCAACC-3´	XM_038680201.1	1001-1021	5	149 bp
IL1RL1_rev	5´-CCAAAGCAAGCAGAGCAGAG-3´	XM_038680201.1	1149-1130	7	
RORC_for	5´-CCACAGAGACAGCACCGAG-3´	XM_038423217.1	83-101	1	148 bp
RORC_rev	5´-AACCCTTGCATCCCTCACAG-3´	XM_038423217.1	230-211	3/4	
RUNX3_for	5´-CAGGTTCAATGACCTCCGCT-3´	XM_847637.3	1965-1984	5	160 bp
RUNX3_rev	5´-CTTCTGCCGGTGTCGTCTG-3´	XM_847637.3	2124-2106	6/7	
TBX21_for	5´-AGAGACCCAGTTCATTGCCG-3´	XM_038675209.1	885-904	4	133 bp
TBX21_rev	5´-CACGCTGGTGTCAACAGATG-3´	XM_038675209.1	1017-998	6	
ZBTB7B_for	5´-GAGGACAGGACCGGAGCA-3´	XM_005622774.1	46-63	1/2	196 bp
ZBTB7B_rev	5´-GCTGTTCATTGAGGCAGCTC-3´	XM_005622774.1	241-222	3	

*ACCN, accession number.

### 2.6 Analysis of canine TCRαβ^+^ T cell subpopulations by flow cytometry

Viability-dye and surface staining of stimulated canine PBMC were essentially performed as described in paragraph 2.3, except for using flow cytometry buffer (3% FBS, 0.1% sodium azide in PBS) instead of PBS/3% FBS. Primary antibodies used are listed in **Table 1**. For intracellular staining of IL-4, the FoxP3 staining kit (Thermo Fisher Scientific) was used according to the manufacturer’s protocol. For intracellular detection of IL-10, alternatively, cells were fixed with 2% paraformaldehyde (Serva, Heidelberg, Germany) for 15 minutes at 4°C and subsequently permeabilized using 0.5% saponin (w/v, Serva) in flow cytometry buffer. After permeabilization, blocking of Fc receptors was performed by incubation with a mixture of rat, mouse and dog serum (each 15% in PBS) for 10 minutes at room temperature. Cells were incubated for 30 minutes at room temperature with the monoclonal antibodies specific for the selected cytokines (**Table 1**). Finally, cells were incubated for 20 minutes with PE-Cy7-conjugated streptavidin to detect IL-4 and IL-10, respectively. Data were acquired with a BD LSR Fortessa (Becton Dickinson, Heidelberg, Germany). FlowJo 10.7.1 software (FlowJo, LLC, Ashland, OR, USA) was used for analysis. Gates were set using appropriate controls: unstimulated controls, and fluorescence minus one (FMO) controls, which include all specific antibodies of the staining panel, except the one of interest, which is replaced by its isotype control.

### 2.7 Statistical analysis

Statistical analysis was performed using Graph Pad Prism 10.2.0 software (San Diego, CA, USA). Depending on normal distribution determined using the Shapiro-Wilk test, data sets are presented with the mean or median. Statistical analysis was performed using One-way ANOVA with Tukey’s post-test or Kruskal-Wallis test with Dunn’s post-test to compare for differences between T cell subpopulations. Additionally, significance of stimulation-induced effects was calculated for each subpopulation by direct comparison of medium (M) versus the PMA/ionomycin-stimulated (P/I) equivalent using the unpaired t test or Mann-Whitney test (two-tailed). The level of confidence for significance is shown in figure legends.

## 3 Results

### 3.1 RT-qPCR analysis of sorted canine conventional and non-conventional TCRαβ^+^ T cell subpopulations demonstrates stability of the CD4^-^CD8α^-^ double-negative phenotype upon activation

For a detailed investigation, non-conventional TCRαβ^+^ CD4^+^CD8α^+^ double-positive (dp) and CD4^-^CD8α^-^ double-negative (dn) T cells were compared to conventional CD4^+^ and CD8α^+^ single-positive (sp) T cells. All four subpopulations were isolated with high purity (>99%) from peripheral blood of healthy Beagle dogs by fluorescence-activated cell sorting (**Figure 1A**) and gene expression was analyzed by reverse transcription-quantitative PCR (RT-qPCR). Noteworthy, a very high transcriptional activity of the dp T cell subset was observed. Despite a >20x lower cell yield of the dp T cell subset compared to conventional T cell subpopulations, the resulting amount of RNA was similar in all groups (**Table 3**), which allowed comparison of gene expression between the populations.

**Table 3:** RNA yield from sorted T cell populations: The low cell number of dp T cells is compensated by their high transcriptional activity.

Sorted TCRαβ^+^ T cell subpopulation	CD4^+^ sp	CD8α^+^ sp	dp	dn
Average* cell number (x 10^5^) used for RNA extraction	9.97 ± 2.3	5.56 ± 0.99	0.21 ± 0.05	3.72 ± 0.66
Average* RNA yield [ng]	638 ± 109	493 ± 83	193 ± 43	327 ± 30
Calculated average* RNA yield per 10^5^ cells [ng]	87 ± 34	84 ± 18	1066 ± 254	102 ± 22

*mean ± SEM (5 individual dogs analyzed in 5 independent experiments).

The transcription of selected key markers was analyzed *ex vivo* (**Figures 1B–D**; **Supplementary Figures 1B–D**) and after stimulation with PMA/Ionomycin (P/I) (**Figures 1E–G**; **Supplementary Figures 1E–G**). In general, gene expression was normalized to the expression levels of two stable reference genes (**Supplementary Table 1**) of different functional classes, *B2M* (**Figures 1**–**4**) and *SDHA* (**Supplementary Figures 1**–**4**), yielding comparable results. The expression of CD4 and *CD8A* mRNA *ex vivo* confirmed the sp/dp/dn phenotype of the sorted T-cell fractions (**Figure 1B**; **Supplementary Figure 1B**). Increased *IL2RA* (CD25) transcription levels *ex vivo* support the high constitutive activation and/or the IL-2 dependence of both non-conventional T cell subpopulations (**Figure 1C**; **Supplementary Figure 1C**) (5, 13). Upregulation of *IL2RA* (CD25) mRNA expression following P/I incubation confirmed efficient stimulation of each T cell subpopulation (**Figure 1F**; **Supplementary Figure 1F**).

In accordance with previous flow cytometric results (13), dp T cells differ from their CD8α^+^ sp counterparts by only weak constitutive transcription of *CD8B* (**Figure 1C**; **Supplementary Figure 1C**), indicating preferential expression of the CD8αα homodimer. After P/I stimulation, *CD8B* was almost not detectable in dp T cells, whereas in CD8α^+^ sp T cells a downregulation was observed (**Figure 1F**; **Supplementary Figure 1F**).

Consistent with their CD4^+^CD8A^+^ phenotype (**Figure 1B**; **Supplementary Figure 1B**), canine peripheral blood dp T cells were characterized by constitutive mRNA expression of genes encoding the transcription factors ThPOK [known to be associated with the CD4 T helper cell gene program (21)] and Runx3 [establishing the gene program characteristic of CD8 cytotoxic T cells (22)] (**Figure 1D**). Albeit showing broader variation among the five dogs studied, ThPOK and Runx3 expression was significantly elevated in dp in comparison to the CD8α^+^ sp and CD4^+^ sp T cell subpopulation, respectively. Dn T cells showed a comparable transcription level of *ZBTB7b* (encoding ThPOK) and *RUNX3* like their CD4^+^ sp counterparts (**Figure 1D**; **Supplementary Figure 1D**). The expected downregulation of *CD4* or *CD8A* mRNA expression in sp and dp populations upon P/I stimulation (5) (**Figure 1E**; **Supplementary Figure 1E**) was accompanied by a downregulation of *ZBTB7b*/*RUNX3* (**Figure 1G**; **Supplementary Figure 1G**). Notably, the CD4^-^CD8α^-^ phenotype of the double-negative T cell population was unaltered upon stimulation (**Figure 1E**; **Supplementary Figure 1E**).

Overall, RT-qPCR analysis of sort-purified canine TCRαβ^+^ T cell subpopulations *ex vivo* and after stimulation confirmed key features of non-conventional dp and dn T cells identified by flow cytometry in previous studies and revealed stability of the dn phenotype upon stimulation *in vitro*.

### 3.2 Canine CD4^+^CD8α^+^ double-positive T cells show a comparable ability to transcribe *IL17A* as CD4^-^CD8α^-^ double-negative T cells and the expected high potential for *IFNG* expression

Expression of characteristic markers of T helper (Th)1 and Th17 cells was analyzed by RT-qPCR in sorted non-conventional and conventional TCRαβ^+^ T cell subpopulations. Consistent with previous flow cytometric results showing expression of the transcription factor T-bet only in a small proportion of dp T cells with a CD4^bright^/CD8α^bright^ phenotype (7), mRNA levels of the corresponding gene *TBX21* was significantly lower in this population as compared to CD8α^+^ sp T cells (**Figure 2A**; **Supplementary Figure 2A**). Upon stimulation, high transcription of *IFNG* (encoding the effector cytokine IFN-γ) was confirmed in both populations (**Figure 2C**; **Supplementary Figure 2C**) (5, 13). Albeit to a lesser extent than in CD8α^+^ sp and dp T cells, a stimulation-dependent increase in transcription of *IFNG* was also observed in CD4^+^ sp and dn T cells (**Figure 2C**; **Supplementary Figure 2C**), consistent with previous flow cytometric results (5). Expression of *RORC*, which encodes the lineage-defining transcription factor of Th17 cells RORγt (23, 24), was hardly detectable in any of the sorted populations *ex vivo* (**Figure 2B**; **Supplementary Figure 2B**) and upon stimulation (data not shown). Nevertheless, transcription of the gene encoding the Th17 effector cytokine IL-17A was highly upregulated in CD4^+^ sp T cells and in dn T cells upon PMA/Iono stimulation (**Figure 2D**; **Supplementary Figure 2D**). This is in line with previous flow cytometric findings (5). Interestingly, high stimulation-induced upregulation of *IL17A* mRNA expression was also found in dp T cells (**Figure 2D**; **Supplementary Figure 2D**) suggesting additional Th17 properties in this population.

### 3.3 Canine CD4^+^CD8α^+^ double-positive and particularly CD4^-^CD8α^-^ double-negative T cells have remarkable T helper 2-like features

To test for a potential role of non-conventional dn and dp T cells in type 2 immune reactions, characteristic features of conventional Th2 cells, including mRNA expression of genes encoding the master transcription factor GATA-3, the key surface marker IL-33Rα as well as the effector cytokines IL-4, IL-5 and IL-13, were analyzed. In line with previous flow cytometric results (5), CD4^+^ sp T cells constitutively expressed *GATA3* and minimal expression was also detected in CD8α sp T cells (**Figure 3A**; **Supplementary Figure 3A**). Furthermore, both, dp and dn T cells constitutively transcribed *GATA3* (**Figure 3A**; **Supplementary Figure 3A**). For dn T cells, this is consistent with previously published flow cytometric results showing an even higher percentage of GATA-3^+^ cells in the dn than in the CD4^+^ sp subpopulation (5). For dp T cells, this is the first indication of a role in type 2 immunity. Indeed, dp T cells showed a high capacity for transcription of genes encoding the Th2-associated cytokines IL-4 and IL-13 comparable to their CD4^+^ sp counterparts (**Figure 3B**; **Supplementary Figure 3B**). However, in dp T cells and their CD4^+^ sp counterparts, stimulation-induced transcription levels of the gene encoding IL-5 were not increased similarly as in CD8α^+^ sp T cells (**Figure 3B**; **Supplementary Figure 3B**). Strikingly, highest mRNA expression levels of all three type 2 effector cytokines IL-4, IL-5, and IL-13 were detected in dn T cells upon PMA/ionomycin stimulation (**Figure 3B**; **Supplementary Figure 3B**). Constitutive transcription of *IL1RL1* (encoding IL33Rα) in this population further supports the Th2-like phenotype (**Figures 3C, D**; **Supplementary Figures 3C, D**).

Taken together, canine non-conventional dp and especially dn T cells show characteristics of conventional Th2 cells and their high ability to transcribe key effector cytokines points to an important involvement in type 2 immune responses.

### 3.4 Canine CD4^-^CD8α^-^ double-negative T cells constitutively express key regulatory markers and show stimulation-induced up-regulation of inhibitory molecules

In a previous study, we identified expression of the master transcription factor of conventional CD4^+^ regulatory T (Treg) cells FoxP3 in a subset of dn T cells (5). Here, we further assessed the potential immunoregulatory phenotype of canine non-conventional dn and dp T cells. Comparable mRNA expression levels of *FOXP3* and the inhibitory receptor *CTLA4* were observed *ex vivo* in sorted dn, dp, and conventional CD4^+^ sp T cells (**Figures 4A, B**; **Supplementary Figures 4A, B**). Interestingly, the gene encoding the immunosuppressive cytokine IL-10 was constitutively transcribed by dn and CD4^+^ sp but not dp T cells (**Figure 4C**; **Supplementary Figure 4C**). High up-regulation of *IL10* and *CTLA4* mRNA expression upon stimulation was detected in CD4^+^ sp but not CD8α^+^ sp and dp T cells (**Figures 4D, E**; **Supplementary Figures 4D, E**). Intriguingly, dn T cells showed highest stimulation-induced expression levels of both inhibitory molecules, which even exceeded those of conventional CD4^+^ T cells (**Figures 4D, E**; **Supplementary Figures 4D, E**). Taken together, these data point to a putative regulatory role of canine non-conventional dn T cells.

### 3.5 Canine CD4^-^CD8α^-^ double-negative T cells are potent IL-4 and IL-10 producers

**Figure 5:**
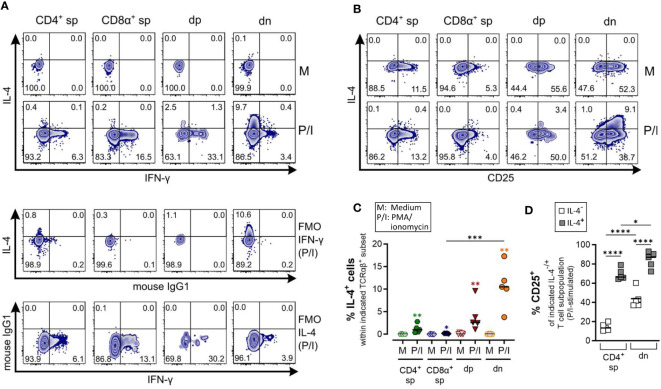
Canine CD4^-^CD8α^-^ double-negative T cells are potent IL-4 producers upon PMA/Ionomycin stimulation. **(A–D)** Whole PBMC of five dogs analyzed in three independent experiments were incubated for 5 h in medium (M) or stimulated with PMA/ionomycin (P/I) in the presence of Brefeldin A and intracellular cytokine staining was performed. **(A)** Representative zebra plots illustrating IL-4 and IFN-γ expression by canine CD4^+^ single-positive (sp), CD8α^+^ sp, CD4^+^CD8α^+^ double-positive (dp) and CD4^-^CD8α^-^ double-negative (dn) T cell subpopulations gated as in **Figure 1A**. Appropriate cytokine gates were set using unstimulated control (medium), and fluorescence minus one (FMO) controls. The dn population shows the highest frequency of IL-4-producing cells upon stimulation. **(B)** Representative zebra diagrams show that upon stimulation, IL-4 is primarily produced by dn T cells with an activated CD25^+^ phenotype. **(C)** Quantification of IL-4^+^ cells within the indicated TCRαβ^+^ subpopulations after 5 h of medium (M) incubation or stimulation with PMA/ionomycin (P/I). Horizontal bars indicate median values. Statistical differences between subpopulations are marked with lines. Additionally, statistical significance of stimulation-induced effects was calculated for each subpopulation by direct comparison of M versus the P/I equivalent (colored asterisks without lines) (* p < 0.05; ** p < 0.01; *** p < 0.001). **(D)** The frequency of CD25^+^ cells within the IL-4^-^ and IL-4^+^ fraction of P/I-stimulated CD4^+^ sp and dn populations was quantified. The horizontal bars indicate mean values. (* p < 0.05; **** p < 0.0001). (CD8α^+^ sp and dp populations were excluded from this analysis due to limiting absolute numbers of IL-4^+^ cells).

The capacity of dp and dn T cells to produce IL-4 and IL-10 was analyzed at the protein level by flow cytometry. Analysis of IFN-γ by intracellular cytokine staining (ICS) served as a positive control. As expected (7), IFN-γ was detected in all TCRαβ^+^ T cell populations analyzed after 5 h of PMA/Iono stimulation, with highest frequencies in dp and CD8α^+^ T cells, followed by CD4^+^ sp and dn T cells (**Figure 5A**).

**Figure 6:**
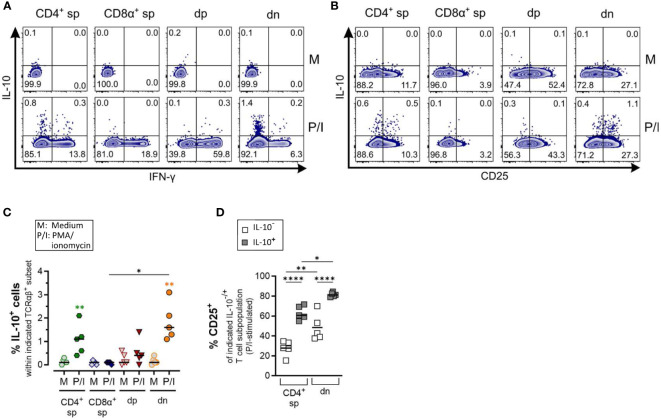
Canine CD4^-^CD8α^-^ double-negative T cells produce IL-10 upon stimulation **(A)** Whole PBMC were incubated for 5 h in medium (M) or stimulated with PMA/ionomycin (P/I) in the presence of Brefeldin A and intracellular cytokine staining was performed. **(A)** Representative zebra plots illustrating IL-10 and IFN-γ expression by canine CD4^+^ single-positive (sp), CD8α^+^ sp, CD4^+^CD8α^+^ double-positive (dp) and CD4^+^CD8α^+^ double-negative (dn) T cell subpopulations gated as in **Figure 1A**. Appropriate cytokine gates were set using the unstimulated control (medium). Dn T cells show the highest frequency of IL-10-producing cells. **(B)** IL-10 producers within dn T cells are mainly CD25^+^. **(C)** Quantification of IL-10^+^ cells within the indicated TCRαβ^+^ subpopulations after 5 h of medium (M) incubation or stimulation with PMA/ionomycin (P/I) (n=5). Horizontal bars indicate median values. Statistical differences between subpopulations are marked with lines. Additionally, statistical significance of stimulation-induced effects was calculated for each subpopulation by direct comparison of M versus the P/I equivalent (colored asterisks without lines) (* p < 0.05; ** p < 0.01). **(D)** The frequency of CD25^+^ cells was quantified within the IL-10^-^ and IL-10^+^ fraction of P/I-stimulated CD4^+^ sp and dn populations with significant induction of IL-10. Horizontal bars indicate mean values. (* p < 0.05; ** p < 0.01, **** p < 0.0001).

Specific detection of IL-4 and IL-10 by ICS upon PMA/Iono stimulation was confirmed using several controls, including unstimulated and fluorescence minus one (FMO) controls. CD8α^+^ sp T cells showed almost no IL-4/IL-10 expression after stimulation (**Figures 5**, **6**).

As expected, the Th1 and Th2 effector cytokines, IFN-γ and IL-4, are barely co-produced (**Figure 5A**). Polyclonal activation-induced IL-4 expression was detected in CD4^+^ sp T cells, and especially in dn T cells. Of note, the frequency of IL-4^+^ cells was higher in the dn than in the CD4^+^ sp population (**Figures 5A, C**). Furthermore, IL-4^+^ dn and CD4^+^ sp T cells exhibit an activated phenotype, indicated by CD25 expression (**Figures 5B, D**).

Production of the immunosuppressive cytokine IL-10 was significantly induced in dn T cells and CD4^+^ sp (but not in CD8α^+^ sp and dp T cells) in response to PMA/Ionomycin stimulation (**Figures 6A–C**). As expected, the majority of dn T cells producing IL-10 are CD25^+^ reminiscent of a T regulatory phenotype (**Figure 6D**).

Taken together, ICS analysis confirmed the high ability of dn T cells to produce IL-4 and IL-10, further supporting a potential role of these non-conventional T cells in type 2 and regulatory immune responses.

## 4 Discussion

Despite indications of their *in vivo* relevance, e.g. in the context of desensitization of dogs with food hypersensitivity (16), canine non-conventional dp and dn T cells have been poorly characterized. To gain deeper insight into their phenotype(s) and potential function(s), in this study, peripheral blood non-conventional dp and dn T cells were comprehensively analyzed *ex vivo* and *in vitro* upon stimulation in comparison to their conventional CD4 and CD8α^+^ sp counterparts. Besides identification of novel characteristics of canine dp T cells (e.g. Th17 potential), three remarkable novel features of dn T cells were revealed, i.e. (1) the CD4^-^CD8α^-^ dn phenotype appears to be stable during *in vitro* stimulation, (2) dn T cells have a high ability to express Th2-associated factors, and in addition to a putative role in type 2 immunity, (3) canine dn T cells seem to have immunosuppressive capacity indicated by strong expression of regulatory molecules.

A role of canine dn T cells in type 2 immunity was hypothesized based on the previous finding that a significant proportion of these cells express GATA-3 (5), the master transcription factor of conventional Th2 cells (25, 26). To better assess a potential type 2 phenotype of canine dn T cells here, characteristic features of Th2 cells including the ability to transcribe the genes encoding IL-4, IL-5, and IL-13 were investigated. These effector cytokines are associated with distinct functions *in vivo*: IL-4 is critical for induction of IgE production, IL-5 mediates recruitment of eosinophils, and IL-13 induces goblet cell hyperplasia, mucus production, and smooth muscle contraction (27). In line with *GATA3* expression, dn T cells showed highest stimulation-induced mRNA expression of *IL4*, *IL5*, and *IL13*. Their strong ability to produce IL-4 was confirmed at the protein level. Noteworthy, upon stimulation, the percentage of IL-4-producing cells was even higher in the non-conventional dn compared to the conventional CD4^+^ sp population. This supports an important role of canine GATA-3^+^ dn T cells in type 2 immunity, like anti-parasite responses and/or allergy. A potential involvement of murine TCRαβ^+^ dn T cells in type 2 immune responses has been suggested in a single study, showing that murine splenic TCRαβ^+^ dn T cells are able to produce high amounts of IL-4 upon activation (28). Interestingly, TCRαβ^+^ dn T cells of healthy humans hardly secrete IL-4 and only some IL-5 upon stimulation (14). The distinct type 2 phenotype of canine non-conventional dn T cells identified in this study was further supported by their constitutive transcription of the gene encoding the IL-33Rα. IL-33 is a well-known key mediator to promote Th2-associated immunity exerting its biological effects via the IL-33R (29). Thus, the impact of IL-33 on the effector functions of canine dn T cells will be an interesting aspect to further investigate in future experiments. Noteworthy, some features of Th2 cells, i.e. transcription of *GATA3* and stimulation-dependent up-regulation of IL-4 and *IL13* expression were also identified in non-conventional dp T cells in the present study. Similar to human allergic disease, canine allergy is characterized by increased expression of the type 2 cytokines IL-4, IL-5 and IL-13, e.g. in dogs with atopic dermatitis (30–32). In light of our present *in vitro* data it will be interesting to evaluate whether canine dn T cells are a major cellular source of type 2 cytokines also *in vivo* and contribute to the pathogenesis of allergic disease.

Besides a potential role in type 2 immunity, canine non-conventional dn T cells are likely involved in immune regulation: In accordance with expression of FoxP3 already shown previously by us (5), we demonstrated a high capacity of dn T cells to transcribe the co-inhibitory receptor CTLA-4 and to produce the immunosuppressive cytokine IL-10 upon stimulation here. Interestingly, the increase of peripheral blood dn T cells in dogs with adverse food reaction after specific immunotherapy could be based on an immunoregulatory role of dn T cells *in vivo* (16). However, FoxP3 expression as well as immunosuppressive effector molecules were not analyzed in this study. Pinheiro et al., already suspected but did not analyze a possible regulatory function of canine FoxP3^+^ dn T cells (33). Noteworthy, albeit murine and human dn T cells lack FoxP3 expression (14, 34–36), they were shown to control immune responses both *in vitro* and *in vivo* and thus have been termed dn regulatory T cells (14, 37–40). Therefore, it can be speculated that in dogs both, FoxP3^+^ and FoxP3^-^ regulatory T cells exist. Their actual suppressive capacity needs to be assessed *in vitro* and *in vivo* in future experiments.

Noteworthy, in canine dp T cells, in contrast to their conventional CD4^+^ sp and dn counterparts, *FOXP3* mRNA expression is not associated with constitutive transcription of *IL10*. Additionally, only weak induction of *IL10* transcription comparably low as in the CD8α^+^ sp population and no upregulation of *CTLA4* mRNA expression was observed in dp T cells upon stimulation. Whereas FoxP3 is a distinct marker of murine regulatory CD4^+^ T cells, in humans, activated non-regulatory CD4^+^ T cells were shown to also express FoxP3 albeit transiently, and at lower levels than human regulatory CD4^+^ T cells (41, 42). In future experiments, the investigation of further regulatory effector molecules and mechanisms will shed light onto the actual regulatory capacity of dp T cells.

The diverse immunological potential of dp T cells was further highlighted in this study showing induction of mRNA expression of various T helper cell-associated cytokines (i.e., *IFNG, IL17A, IL4, IL13*) upon stimulation. The increased transcriptional activity of canine dp T cells shown here represents an important prerequisite for future single-cell RNA sequencing analyses, that could shed light on their potential polyfunctionality, i.e. their ability to produce combinations of cytokines at the single-cell level. Furthermore, scRNA-seq including TCR repertoire analysis, recently established for total canine TCRαβ^+^ T cells by our group (43), should be applied to both, purified dp and dn T cells in future experiments as this could provide initial insights into a potential biased recognition of antigens.

Taken together, the data presented here reveal new insights into phenotypic features and potential functions of canine dp and dn T cells and provide the basis for future *in vitro* and *in vivo* studies to elucidate their role in host defense and immunopathological diseases of dogs.

## Data availability statement

The original contributions presented in the study are included in the article/**Supplementary Material**. Further inquiries can be directed to the corresponding author.

## Ethics statement

The animal study was approved by Saxony State Office (Landesdirektion Sachsen), Leipzig, Germany (approval numbers: DD24.1-5131/444/30; DD24.1-5131/468/16). The study was conducted in accordance with the local legislation and institutional requirements.

## Author contributions

MP: Data curation, Formal analysis, Investigation, Methodology, Visualization, Writing – original draft, Writing – review & editing. DD: Data curation, Formal analysis, Investigation, Methodology, Writing – review & editing. PM: Resources, Writing – review & editing. MB: Conceptualization, Writing – review & editing. GA: Conceptualization, Funding acquisition, Resources, Supervision, Writing – review & editing. ME: Conceptualization, Data curation, Formal analysis, Funding acquisition, Investigation, Methodology, Project administration, Supervision, Visualization, Writing – original draft, Writing – review & editing.
