# Supplementary material for: Canine peripheral non-conventional TCRαβ+ CD4-CD8α- double-negative T cells show T helper 2-like and regulatory properties
Source: Front Immunol. 2024 May 21;15:1400550. doi: 10.3389/fimmu.2024.1400550 (PMC11148280; doi:10.3389/fimmu.2024.1400550)
Supplement: Supplementary file 1 [file Presentation_1.pptx]

## Slide 1
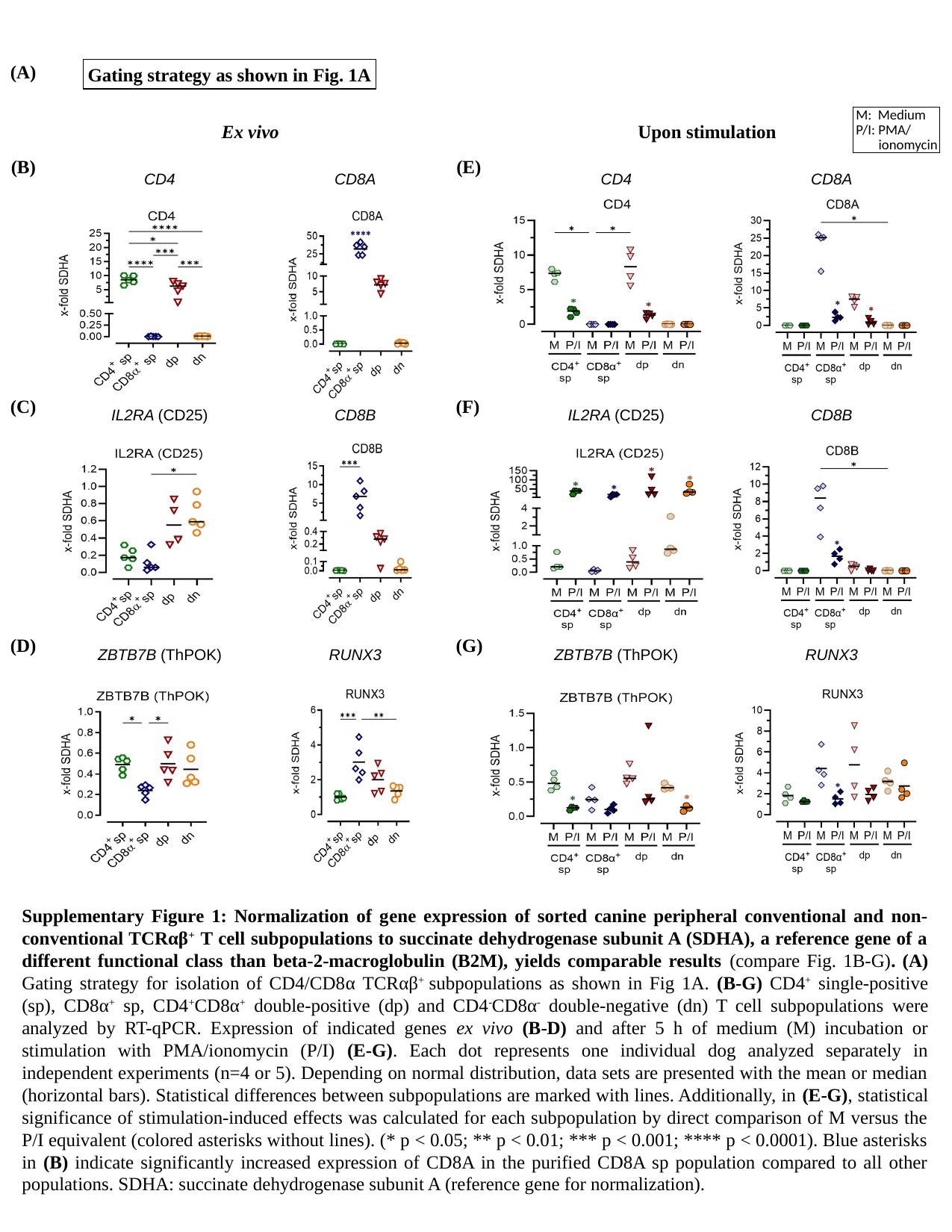

(A)
Gating strategy as shown in Fig. 1A
M: Medium
P/I: PMA/
 ionomycin
Ex vivo
Upon stimulation
(B)
(E)
CD4
CD8A
CD4
CD8A
(C)
(F)
IL2RA (CD25)
CD8B
IL2RA (CD25)
CD8B
(D)
(G)
ZBTB7B (ThPOK)
RUNX3
ZBTB7B (ThPOK)
RUNX3
Supplementary Figure 1: Normalization of gene expression of sorted canine peripheral conventional and non-conventional TCRαβ+ T cell subpopulations to succinate dehydrogenase subunit A (SDHA), a reference gene of a different functional class than beta-2-macroglobulin (B2M), yields comparable results (compare Fig. 1B-G). (A) Gating strategy for isolation of CD4/CD8α TCRαβ+ subpopulations as shown in Fig 1A. (B-G) CD4+ single-positive (sp), CD8α+ sp, CD4+CD8α+ double-positive (dp) and CD4-CD8α- double-negative (dn) T cell subpopulations were analyzed by RT-qPCR. Expression of indicated genes ex vivo (B-D) and after 5 h of medium (M) incubation or stimulation with PMA/ionomycin (P/I) (E-G). Each dot represents one individual dog analyzed separately in independent experiments (n=4 or 5). Depending on normal distribution, data sets are presented with the mean or median (horizontal bars). Statistical differences between subpopulations are marked with lines. Additionally, in (E-G), statistical significance of stimulation-induced effects was calculated for each subpopulation by direct comparison of M versus the P/I equivalent (colored asterisks without lines). (* p < 0.05; ** p < 0.01; *** p < 0.001; **** p < 0.0001). Blue asterisks in (B) indicate significantly increased expression of CD8A in the purified CD8A sp population compared to all other populations. SDHA: succinate dehydrogenase subunit A (reference gene for normalization).

## Slide 2
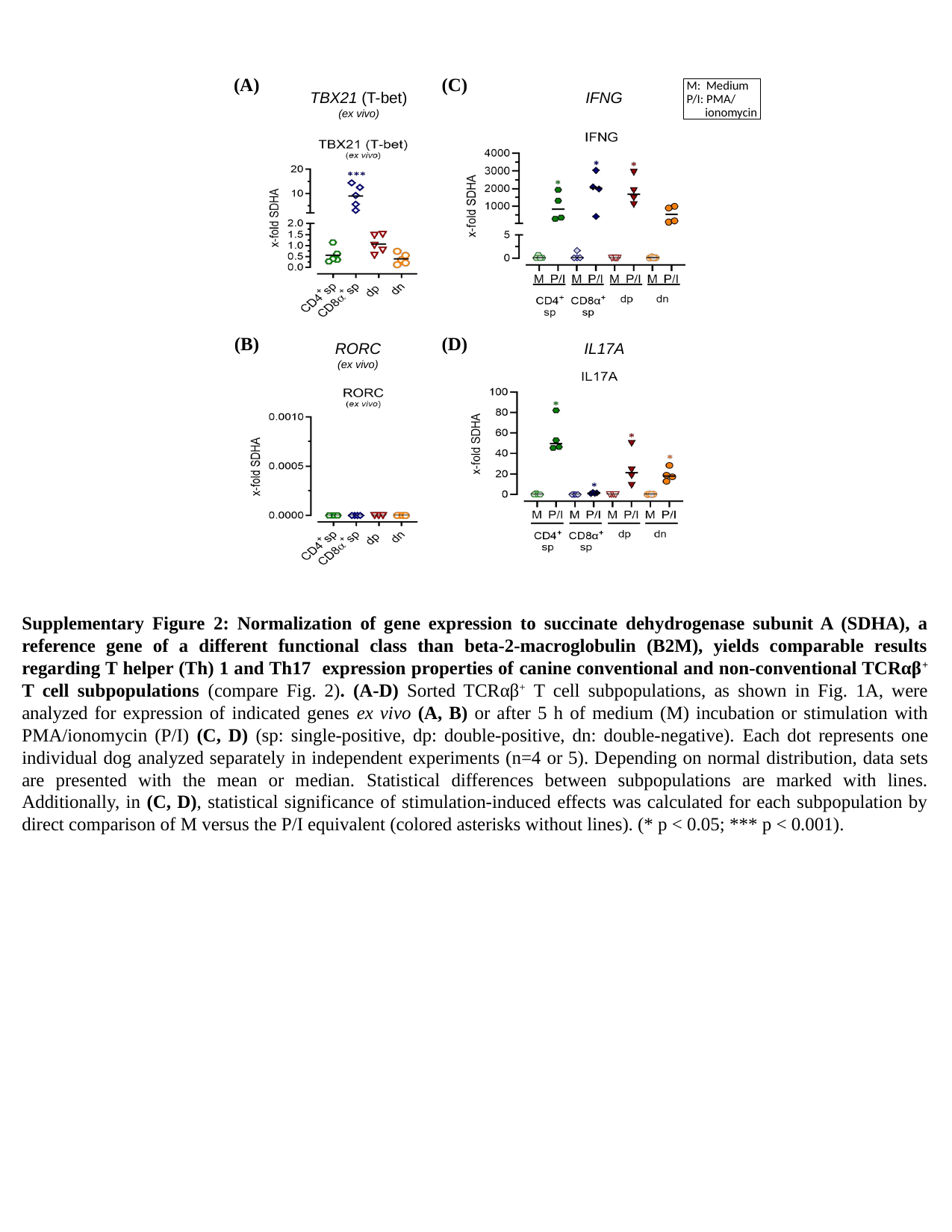

(A)
(C)
M: Medium
P/I: PMA/
 ionomycin
TBX21 (T-bet)
(ex vivo)
IFNG
(B)
(D)
RORC
(ex vivo)
IL17A
Supplementary Figure 2: Normalization of gene expression to succinate dehydrogenase subunit A (SDHA), a reference gene of a different functional class than beta-2-macroglobulin (B2M), yields comparable results regarding T helper (Th) 1 and Th17 expression properties of canine conventional and non-conventional TCRαβ+ T cell subpopulations (compare Fig. 2). (A-D) Sorted TCRαβ+ T cell subpopulations, as shown in Fig. 1A, were analyzed for expression of indicated genes ex vivo (A, B) or after 5 h of medium (M) incubation or stimulation with PMA/ionomycin (P/I) (C, D) (sp: single-positive, dp: double-positive, dn: double-negative). Each dot represents one individual dog analyzed separately in independent experiments (n=4 or 5). Depending on normal distribution, data sets are presented with the mean or median. Statistical differences between subpopulations are marked with lines. Additionally, in (C, D), statistical significance of stimulation-induced effects was calculated for each subpopulation by direct comparison of M versus the P/I equivalent (colored asterisks without lines). (* p < 0.05; *** p < 0.001).

## Slide 3
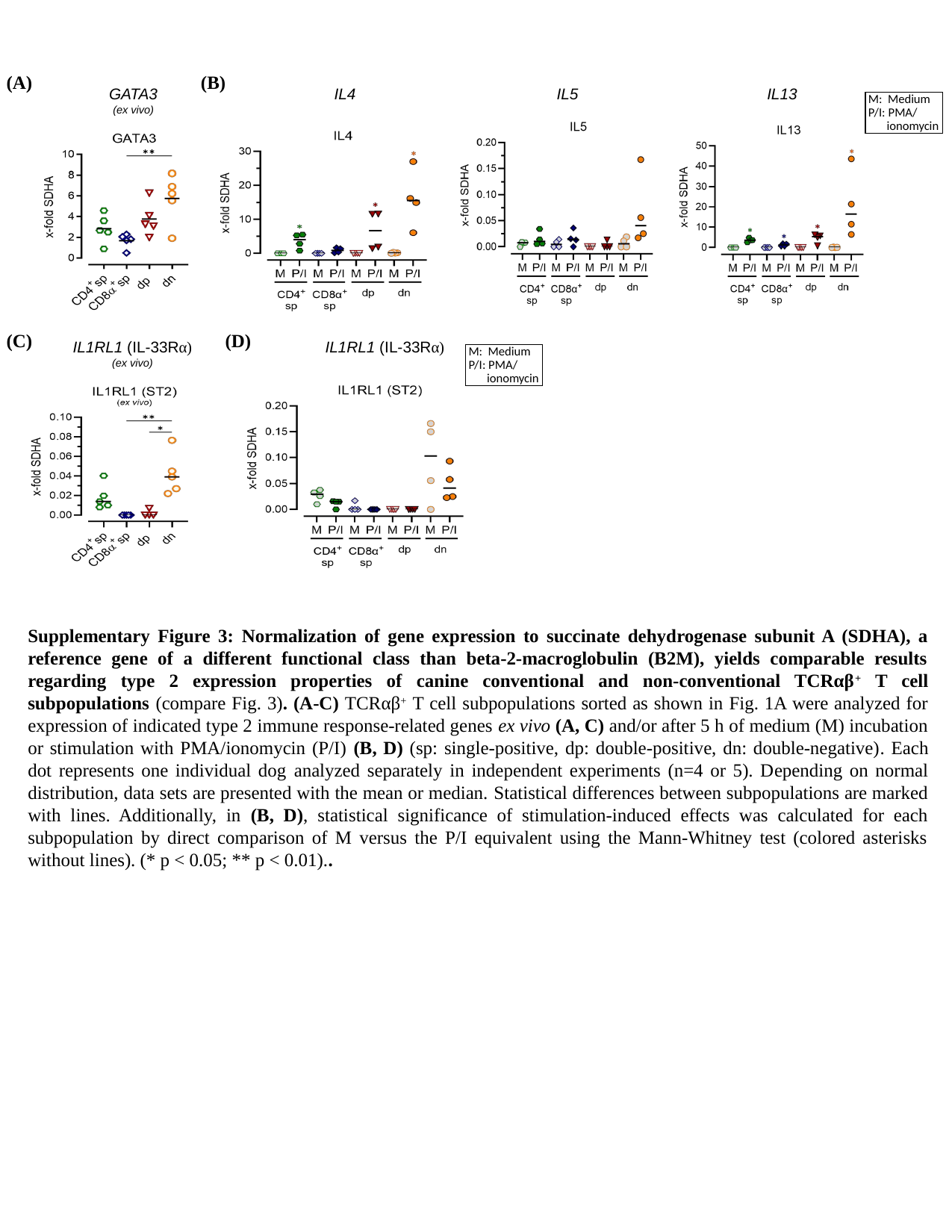

(B)
(A)
GATA3
(ex vivo)
IL4
IL5
IL13
M: Medium
P/I: PMA/
 ionomycin
(C)
(D)
IL1RL1 (IL-33Rα)
(ex vivo)
IL1RL1 (IL-33Rα)
M: Medium
P/I: PMA/
 ionomycin
Supplementary Figure 3: Normalization of gene expression to succinate dehydrogenase subunit A (SDHA), a reference gene of a different functional class than beta-2-macroglobulin (B2M), yields comparable results regarding type 2 expression properties of canine conventional and non-conventional TCRαβ+ T cell subpopulations (compare Fig. 3). (A-C) TCRαβ+ T cell subpopulations sorted as shown in Fig. 1A were analyzed for expression of indicated type 2 immune response-related genes ex vivo (A, C) and/or after 5 h of medium (M) incubation or stimulation with PMA/ionomycin (P/I) (B, D) (sp: single-positive, dp: double-positive, dn: double-negative). Each dot represents one individual dog analyzed separately in independent experiments (n=4 or 5). Depending on normal distribution, data sets are presented with the mean or median. Statistical differences between subpopulations are marked with lines. Additionally, in (B, D), statistical significance of stimulation-induced effects was calculated for each subpopulation by direct comparison of M versus the P/I equivalent using the Mann-Whitney test (colored asterisks without lines). (* p < 0.05; ** p < 0.01)..

## Slide 4
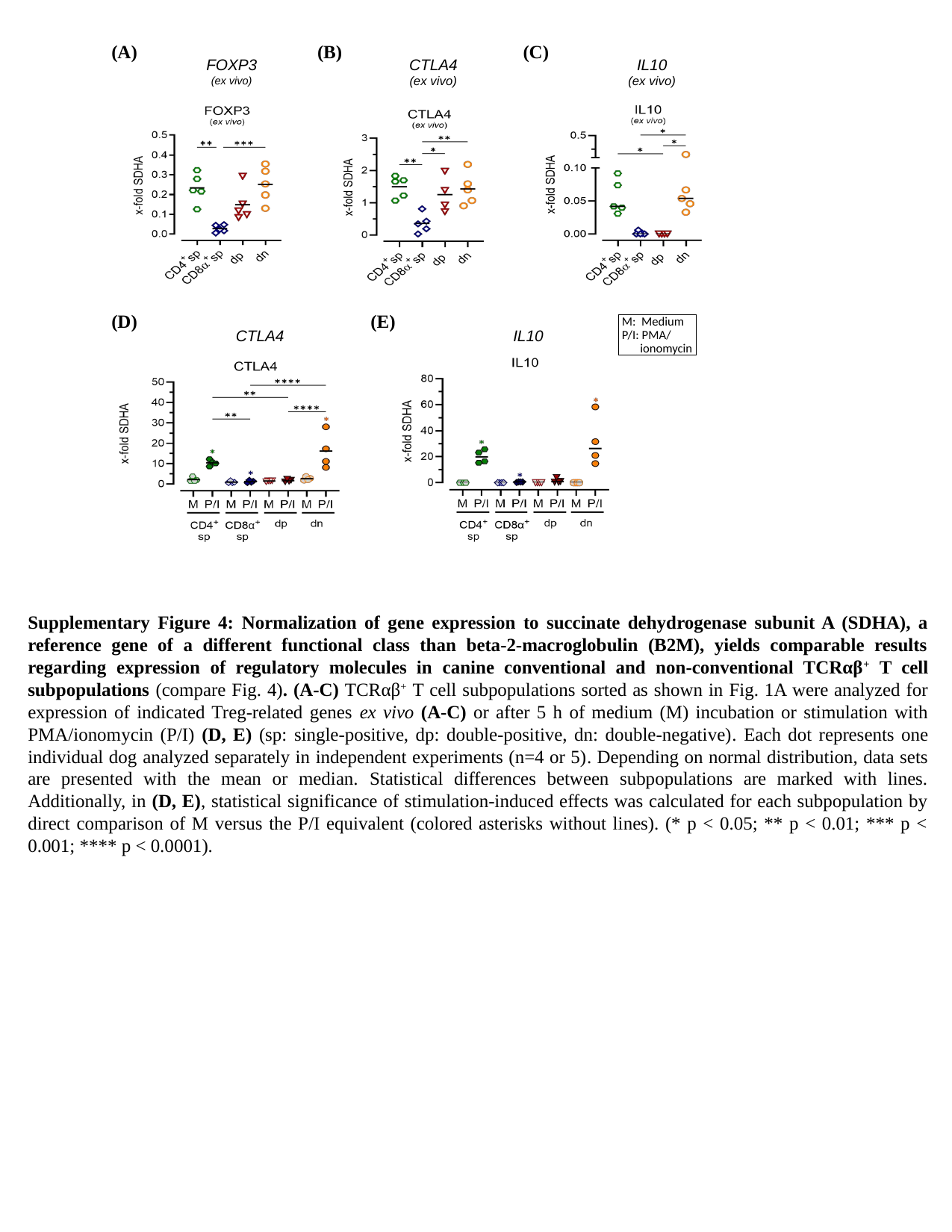

(A)
(B)
(C)
FOXP3
(ex vivo)
CTLA4
(ex vivo)
IL10
(ex vivo)
(D)
(E)
M: Medium
P/I: PMA/
 ionomycin
CTLA4
IL10
Supplementary Figure 4: Normalization of gene expression to succinate dehydrogenase subunit A (SDHA), a reference gene of a different functional class than beta-2-macroglobulin (B2M), yields comparable results regarding expression of regulatory molecules in canine conventional and non-conventional TCRαβ+ T cell subpopulations (compare Fig. 4). (A-C) TCRαβ+ T cell subpopulations sorted as shown in Fig. 1A were analyzed for expression of indicated Treg-related genes ex vivo (A-C) or after 5 h of medium (M) incubation or stimulation with PMA/ionomycin (P/I) (D, E) (sp: single-positive, dp: double-positive, dn: double-negative). Each dot represents one individual dog analyzed separately in independent experiments (n=4 or 5). Depending on normal distribution, data sets are presented with the mean or median. Statistical differences between subpopulations are marked with lines. Additionally, in (D, E), statistical significance of stimulation-induced effects was calculated for each subpopulation by direct comparison of M versus the P/I equivalent (colored asterisks without lines). (* p < 0.05; ** p < 0.01; *** p < 0.001; **** p < 0.0001).
