# Supplementary material for: Canine peripheral non-conventional TCRαβ+ CD4-CD8α- double-negative T cells show T helper 2-like and regulatory properties
Source: Front Immunol. 2024 May 21;15:1400550. doi: 10.3389/fimmu.2024.1400550 (PMC11148280; doi:10.3389/fimmu.2024.1400550)
Supplement: Supplementary file 2 [file Table_1.docx]

Supplementary Material

**Supplementary Table 1:** M factor determination of candidate reference genes β-2-microglobulin (B2M), ribosomal protein S 19 (RPS19), and succinate dehydrogenase subunit A (SDHA). According to Vandesompele et al., 2002, the candidate reference genes belong to different functional classes to reduce the chance that genes may be co-regulated (17). The expression of B2M, RPS19 and SDHA was compared among the four canine T cell subpopulations under all conditions (*ex vivo*, medium incubation and PMA/Iono stimulation). The determination of the gene stability was carried out by the CFX Maestro software (BioRad, Munich, Germany), which utilizes the GeNorm algorithm (17). Briefly, the M factor is the result of the arithmetic mean of all pairwise variations between the ratios of pairs of reference genes. The lower the M factor, the more stable the reference gene. CFX Maestro considers “ideal” genes with an M factor lower than 0.5, “acceptable” between 0.5 and 1 and “unstable” greater than 1. B2M and SDHA were selected as reference genes in the study.

| Order | Gene Name | Evaluation | Average M Value  (AvgM) | Stability  (Ln(1/AvgM)) | Number of Samples |
| --- | --- | --- | --- | --- | --- |
| 1 | CL_B2M | Ideal | 0.468 | 0.759 | 11 |
| 2 | CL_SDHA | Ideal | 0.468 | 0.759 | 11 |
| 3 | CL_RPS19 | Acceptable | 0.554 | 0.590 | 11 |
